# Dose effects of mycophenolate mofetil in Chinese patients with neuromyelitis optica spectrum disorders: a case series study

**DOI:** 10.1186/s12883-018-1056-x

**Published:** 2018-04-23

**Authors:** Yujuan Jiao, Lei Cui, Weihe Zhang, Chunyu Zhang, Yeqiong Zhang, Xin Zhang, Jinsong Jiao

**Affiliations:** 1 Department of Neurology, China-Japan Friendship Hospital, #2 Yinghuayuan East Street, Chaoyang District, Beijing, 100029 China; 2 Department of Health Reform and Development, China-Japan Friendship Hospital, Beijing, China

**Keywords:** Mycophenolate mofetil, Neuromyelitis optica, Neuromyelitis optica spectrum disorders, Efficacy, Tolerability, Dose effects

## Abstract

**Background:**

Neuromyelitis optica (NMO) spectrum disorder (NMOSD) is a devastating autoimmune inflammatory disorder of the central nervous system, which can result in blindness or paralysis. Currently, there is a dire need for new treatment options in the clinic. Several case series have shown that mycophenolate mofetil (MMF) may be an effective treatment for NMOSD patients. The dosing of MMF in the treatment of NMOSD has been poorly studied. Therefore, we evaluated the efficacy, tolerability, influential factors and optimal dosage of MMF in Chinese patients with NMOSD.

**Methods:**

A case series of 109 NMO or NMOSD (limited forms of NMO with seropositive AQP4-IgG) patients were retrospectively analyzed and followed up. Out of the 109 patients, 86 patients had received MMF for 6 months or longer and were included for efficacy assessment.

**Results:**

When comparing the annualized relapse rate (ARR) of MMF treatment with that of pre-MMF treatment period, MMF was found to significantly reduce ARR in 75 (87%) patients (*p* < 0.0001). The median pre-treatment Expanded Disability Status Scale (EDSS) score in remission decreased from 3 (range, 0–8.5) to 2.5 (range, 0–8) at the last follow-up (*p* = 0.006), yet no significant difference was found in the visual score. The higher doses of MMF (1750 mg/d to 2000 mg/d) significantly lowered the relapse risks compared with lower doses (1000 mg/d or less, *p* < 0.0001) or moderate doses (1250 to 1500 mg/d, *p* = 0.031). Coexisting with systemic autoimmune diseases (HR, 2.418; *p* = 0.0345) and attack number before MMF initiation (HR, 1.117; *p* = 0.02) were important risk factors for relapses. MMF was generally well tolerated with adverse effects occurring in 21 patients (19%). While four patients decreased their daily doses because of the adverse effects, only one patient stopped MMF treatment.

**Conclusions:**

MMF is generally effective and well tolerated in Chinese NMOSD patients. High-dose MMF was more potent than the lower dose for NMOSD patients, with 1750 mg of daily MMF being the recommended dosage for Chinese patients with NMOSD. MMF treatment reduces the frequency of relapses and improves the quality of life for patients with this debilitating disease.

**Electronic supplementary material:**

The online version of this article (10.1186/s12883-018-1056-x) contains supplementary material, which is available to authorized users.

## Background

Neuromyelitis optica (NMO) spectrum disorder (NMOSD) is a devastating autoimmune inflammatory disorder of the central nervous system, which can lead to blindness or paralysis. The risk of developing disabilities increases significantly with the number of relapses [1, 2]. Prevention of relapse is essential for the successful treatment of NMOSD patients. While there have been no placebo-controlled or comparative randomized controlled trials of immunosuppressive therapies conducted in NMO patients, several case series have reported that mycophenolate mofetil (MMF) may be effective for treatment of NMOSD [3–10]. To date, there are no clear recommendations regarding the dosing of MMF. In this study, we aimed to evaluate the efficacy, safety profile and recommendable dosage of MMF in a large cohort of Chinese patients with NMO and NMOSD.

## Methods

### Patients

This study was approved by the Medical Ethics Committee of China-Japan Friendship Hospital (2016–62). Patient consent forms were obtained from all patients or his/her legal representatives before the study. We performed a retrospective review of the medical records from patients that presented with NMO, using the 2006 revised NMO criteria [11] or the NMOSD (limited forms of NMO with seropositive AQP4-IgG) [12]. From January 2009 to October 2016, 109 patients (96 female and 13 males) received MMF treatment and were enrolled for individual tolerability assessments. Of the 109 patients, 86 received ≥6 months of MMF were included for the efficacy assessment, 22 patients had recently initiated MMF treatment, and one patient stopped MMF before the end of 6 months due to an adverse reaction. Patients who received 1000 mg/d or less of MMF were classified as the low-dose treatment group, while MMF dosages of 1250 mg/d and 1500 mg/d were deemed as the moderate-dose treatment. The highest dosages utilized in this study were 1750 mg/d and 2000 mg/d, which were considered as the high-dose treatment. While receiving MMF treatment, each patient received long-term concomitant oral corticosteroids (10–15 mg every other day) for the first one to two years. At each follow-up appointment, routine blood tests were performed to assess the efficacy of the therapy. The patients were recommended to follow-up every 3 months, and there was a minimum annual follow-up requirement and all of the follow-up appointments were recorded.

### Clinical assessment

Data was recorded for the patients, including demographic data, detailed treatment plans (daily dose of MMF and glucocorticoid, date and reason for the initiation or cessation of immunosuppressive agents, and the starting or stopping of any other treatments), clinical course, adverse reactions, modified Expanded Disability Status Scale (EDSS) and corrected visual acuity at remission and each follow-up appointment [1].

Visual acuity was assessed separately for each eye using the following scale: 0 = 20/20; 1 = scotoma, but better than 20/30; 2 = 20/30 to 20/59; 3 = 20/60 to 20/199; 4 = 20/200 to 20/800; 5 = count fingers only; 6 = light perception; 7 = no light perception [1]. The visual outcome in remission was the sum of the visual scores for each eye after each attack and at the follow-up appointments.

A relapse is defined as a sudden worsening of neurological function lasting for more than 24 h that is unknown in origin with no other identifiable causes, such as a fever or infection. Additionally, a relapse will increase the EDSS score by a half point or more, or it may be indicated by a worsening of one point in two of the functional systems or two points in a single functional system. A severe relapse was defined as an EDSS score of six or more, which required a walking aid to travel 100 m with or without resting, at the nadir of the attack. In those patients with baseline EDSS scores ≥6.0, an increase of 0.5 points or more was classified as a severe relapse. In cases of optic neuritis (ON), a severe relapse was defined as a sudden worsening of visual acuity (VA) of 0.1 or less in patients with baseline VA scores of greater than 0.1. When accompanied with MRI evidence of ON, any decrease of VA was regarded as a severe relapse if the baseline vision was light perception, hand motion, or counting fingers [5, 9]. Suboptimal treatment with MMF was defined as 6 months or less of therapy or daily dosages less than the minimal therapeutic dose (1250 mg in adults).

### Statistical analyses

Data were analyzed using SAS version 9.3 (SAS institution Inc., NC, USA). A two-sided *p* ≤ 0.05 was considered statistically significant. The Wilcoxon signed-rank test was used to compare pre-treatment annualized relapse rates (ARR), EDSS, and visual scores with on-treatment indexes. The number of severe attacks that occurred before and during MMF treatment was compared using the Pearson chi-square test. Characteristics were compared among the different MMF dosage groups (i.e., female and male) using the Pearson chi-square test for categorical data and the Kruskal-Wallis H-test for continuous data. The Kaplan-Meier method was used to determine the time to first relapse among different groups, and were then compared using the log-rank test. Hazard ratios (HR) that pertained to the first relapse after the start of MMF treatment were calculated using the Cox proportional hazard model, as follows:

$$ \mathrm{h}\left(\mathrm{t},\mathrm{x}\right)={\mathrm{h}}_0\left(\mathrm{t}\right)\exp \left({\mathrm{\ss}}_1{\mathrm{x}}_1+{\mathrm{\ss}}_2{\mathrm{x}}_2+\dots +{\mathrm{\ss}}_{\mathrm{m}}{\mathrm{x}}_{\mathrm{m}}\right) $$

where t is the first relapse time, and x is the MMF dosage, concomitant with any systemic autoimmune diseases, pre-MMF ARR, pre-MMF EDSS, duration of MMF therapy, duration of pre-MMF, attacks number before MMF initiation, gender, age at onset and serum AQP4-IgG positivity.

## Results

### Baseline demographic and clinical data

The demographic and clinical characteristics of the cohort are summarized in Table 1. Diagnoses at the initiation of MMF therapy were NMO (64), transverse myelitis (9, recurrent in 7), recurrent optic neuritis (3), and NMOSD with other clinical characteristics (10). Among NMOSD patients receiving different dosages of MMF, there were no significant differences between the baseline characteristics including age, female percentage, complete NMO patient percentage, aquaporin-4 antibody positivity, age at disease onset, duration of MMF treatment, treatment-naïve patients, disease duration, attack number, ARR and EDSS before receiving MMF (Additional file 1: Table S1).

**Table 1:** Demographic characteristics of patients who received MMF treatment for six months or longer

Characteristic	Value
Number of Patients	*n* = 86
Current age, median (range), y	53 (15–84)
Female sex, No. (%)	77 (90%)
NMO diagnosis, No. (%)	64 (74%)
NMOSD diagnosis, No. (%)	22 (26%)
Aquaporin-4 antibody positivity, No. (%)	74 (86%)
Age at onset, median (range), y	43 (6–68)
Overall disease duration, median (range), mo	71 (7–535)
Disease duration before receiving MMF, median (range), mo	71 (6–444)
Attack number before receiving MMF, median (range)	5 (1–33)
Duration of MMF treatment, median (range), mo	20 (6–89)
Abnormal autoantibodies^a^, n (%)	39 (45%)
Coexisting with systemic autoimmune diseases	29 (34%)
Concurrent use of prednisone, n (%)	65 (76%)
treatment-naïve patients, n (%)	21 (24%)
Previous immunosuppressive agents:	
Corticosteroids^b^, n (%)	33 (38%)
Azathioprine, n (%)	15 (17%)
Cyclophosphamide, n (%)	4 (5%)
Rituximab, n (%)	2 (2%)
Tacrolimus (FK506)	1 (1%)
Methotrexate	1 (1%)
Previous immunomodulatory therapies:	
β-interferons, n (%)	6 (7%)
hydroxychloroquine sulfate, n (%)	2 (2%)
Mitoxantrones, n (%)	1 (1%)

^a^Autoantibodies refers to rheumatoid factors, antinuclear antibodies, anti–double-stranded DNA antibodies, ribose nuclear proteins, anti-SM antibodies, anti-SSA and anti-SSB antibodies, TPO and TG antibodies

^b^Corticosteroids refers to continuously taking oral prednisone or methylprednisolone for more than 3 months

### Efficacy: MMF therapy significantly reduced ARR of NMOSD

**Fig. 1:**
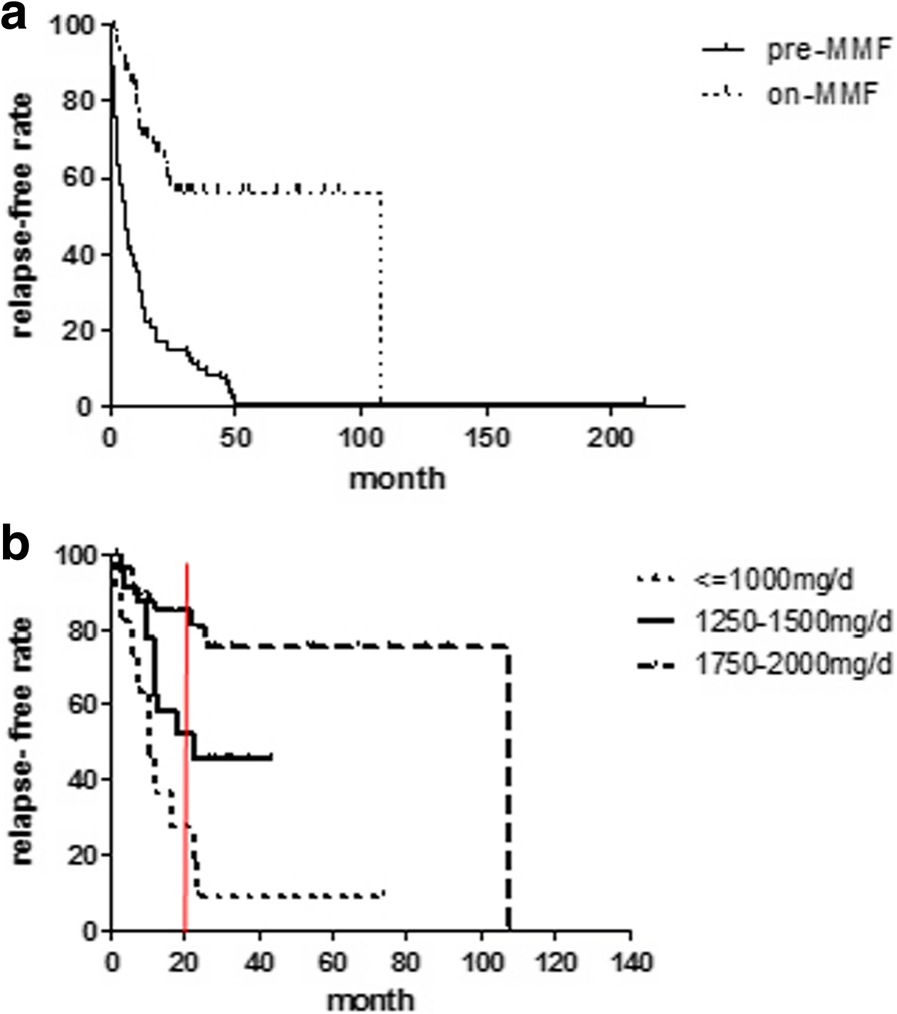
Kaplan-Meier survival estimates pertaining to probabilities of being free of the occurrence of any relapse (**a**) between during MMF treatment (on-MMF, dashed line) and before MMF treatment initiation (pre-MMF, black line) in NMOSD patients (Log-rank test, *p* < 0.001). The relapse-free rates after 1 year and 2 years therapy with MMF were 72% and 58%, respectively. Those values were much higher than 30% and 14% before initiation of MMF therapy. (**b**) with different dose of MMF therapy. After 20 months of MMF therapy, approximately 68% and 42% of NMOSD patients in the high-dose and moderate-dose groups would remain relapse-free, respectively (Log-Rank test, *p* = 0.019). However, only 9% of the patients receiving low-dose MMF would remain relapse-free, significant lower than the moderate or high-dose groups (Log-Rank test *p* = 0.031, *p* < 0.0001)

During a median course of 20 months (average 27) therapy with MMF, 55 (64%) patients were relapse-free and 75 (87%) of the 86 evaluated patients experienced improvement in their ARR. Among the 31 patients who relapsed during MMF therapy, 7 (23%) patients experienced their first relapse within 6 months of initiating MMF therapy. The median ARR during MMF treatment (0, range 0–2.8) was significantly reduced (*p* < 0.0001, Table 2) compared with the pre-treatment ARR (1.4, range 0.1–11.0). The Kaplan-Meier survival estimated the significant difference between the relapse-free rates of pre-treatment and during treatment periods (*p* < 0.001, Fig. 1a).

**Table 2:** Subanalysis of treatment efficacy in patients treated with MMF

	ARR	*p*	EDSS	*P*	On-MMF, Patients, %		
	Median (Range)		Median (Range)		Relapse free	Improved ARR	Improved or Stabilized EDSS
Total patients (*n* = 86)							
Pre-MMF treatment	1.4(0.1–11)	< 0.0001	3(0–8.5)	0.006	64	87	87
on-MMF treatment	0(0–2.8)		2.5(0–8.5)				
Patients with high dose MMF treatment (*n* = 52)							
Pre-MMF treatment	1.5(0.1–11)	< 0.0001	3(0–8.5)	0.511	81	92	89
on-MMF treatment	0(0–2.8)		2.5(0–8.5)				
Patients with moderate dose MMF treatment (*n* = 23)							
Pre-MMF treatment	1.4(0.2–6)	0.0003	3(0–8.5)	0.071	52	78	83
on-MMF treatment	0(0–2.2)		2(0–6.5)				
Patients with low dose MMF treatment (*n* = 11)							
Pre-MMF treatment	1.2(0.5–6)	0.0078	4(0–8.5)	0.438	9	82	91
on-MMF treatment	0.5(0–1.0)		4(0–8.5)				

*pre-MMF* before initiation of MMF treatment, *on-MMF* during treatment of MMF

### Efficacy: MMF therapy significantly decreased the risk of severe relapses

A total of 572 attacks were recorded in 86 patients with NMOSD. Of the 572 attacks, 502 (6 with uncertain severities) of them happened prior to the initiation of MMF therapy, including 200 (40%) attacks rated as severe and 296 attacks rated as mild. During MMF treatment, only 16 (23%) of the 70 relapses were severe. There was a significantly lower risk of patients experiencing severe relapses during MMF treatment when compared with the period prior to MMF therapy initiation (*p* = 0.006).

### Disability efficacy: MMF was effectual for improving disabilities in NMOSD

The EDSS scores improved in 36 patients and were unchanged in 39 patients, which was 75 out of the 86 (87%) NMOSD patients. There was a statistically significant decrease between the median EDSS score obtained at the beginning of MMF treatment (in remission) and at the last follow-up (*p* = 0.006, Table 2). The median visual scores obtained at pre-MMF treatment (in remission) and the last follow-up visit were 2 (average 3.0, range, 0–13) and 1 (average 2.7, range, 0–13), respectively. While the visual scores improved in 11 patients and stabilized in 68 patients (total of 79 out of 86 or 92% NMOSD patients), there was no significant difference between values obtained at pre-treatment and the last follow-up (*P* = 0.106).

### Efficacy: High dose MMF was more potent than lower dose for NMOSD

**Fig. 2:**
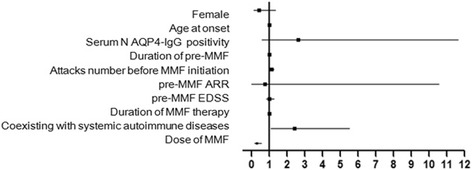
Adjusted Hazard Risks for relapse after MMF initiation, according to the clinical characteristics and based on patients who received MMF for more than 6 months. NMOSD patients treated with the higher dose of MMF had a significantly lower risk of relapse (HR, 0.291; 95% confidence interval, 0.164–0.516; *p* < 0.0001). Significant risk factors for relapse in NMOSD patients were the presence of any systemic autoimmune disease (HR, 2.418; 95% confidence interval, 1.066–5.481; *p* = 0.0345) and an increased number of relapses before MMF initiation (HR, 1.117; 95% confidence interval, 1.018–1.227; *p* = 0.020). Other factors were determined to have little influence on the effect of MMF treatment

Among 31 NMOSD patients who relapsed during MMF therapy, 10 of the 11 (91%) patients were taking the lowest dosage of 1000 mg/d or less MMF relapsed, 11 of the 23 (48%) patients were on the median dosage of 1250 mg/d or 1500 mg/d, and 10 of the 52 patients (19%) were receiving the highest dosage of 1750 mg/d or 2000 mg/d (Table 2). The proportion of patients on concomitant corticosteroids for more than 1 year and 2 years did not differ among the three doses. Statistically significant differences in relapse-free rates were found between the lower and moderate dosage groups (*p* = 0.031), moderate and higher dosage groups (*p* = 0.019), and the lower and higher dosage groups (*p* < 0.0001, Fig. 1b). The adjusted hazard risks also indicated that the higher dosage of MMF was a protective factor for preventing relapse (Fig. 2). The EDSS scores were improved in 18 patients (35%), 13 patients (57%), and 5 patients (46%) in the lower, moderate, and higher MMF dosage groups, respectively. Additionally, the EDSS scores remained unchanged in 28 patients (54%), 6 patients (26%), and 5 patients (46%) in the lower, moderate, and higher MMF dosage groups, respectively. However, among the three MMF dosage groups, there is no statistically significant difference either in the number of patients with improved EDSS scores or in the number of patients with unchanged EDSS scores (*p* = 0.276).

### NMOSD patients coexist with concomitant systemic autoimmune diseases were more prone to relapses

There were 29 (34%) NMOSD patients who had at least one systemic autoimmune disease in this study, which included 20 patients with thyroid disease, 7 patients with Sjögren syndrome, 4 patients with systemic lupus erythematosus, 2 patients with rheumatoid arthritis, 2 patients with Castleman disease, 2 patients with psoriasis, 2 patients with interstitial pneumonia and 1 patient with myasthenia gravis. It was found that concomitant systemic autoimmune disease (HR, 2.418; 95% confidence interval, 1.066–5.481; *p* = 0.0345) and relapses numbers before the initiation of MMF treatment (HR, 1.117; 95% confidence interval, 1.018–1.227; *p* = 0.02) were significant risk factors for the relapse in NMOSD patients (Fig. 2).

### Side-effects and MMF tolerability

Twenty-one of 109 patients (19%) reported adverse effects with MMF treatment, including hair loss (*n* = 5), increased transaminase levels (*n* = 3), low white blood cell and neutrophil counts (*n* = 3, one of these patients also reported interstitial pneumonia and another reported human papillomavirus type 1 [HPV-1] infection), diarrhea and abdominal pain (*n* = 2), shingles (*n* = 2), herpes simplex infection (*n* = 2), headache (*n* = 2), thrombocytopenia (*n* = 1), constipation (*n* = 1), and chronic dermopathy on the hands and nails (n = 1). Five patients (4.6%) reported moderate to severe adverse effects and among them, two patients treated with MMF 2000 mg/d were admitted to hospital due to increased transaminase levels and interstitial pneumonia, respectively. Of the two patients, one discontinued MMF in the first two months of treatment, while the dosage was decreased from 2000 mg/d to 1250 mg/d for the second patient. The dosage of three additional patients was lowered from 2000 mg/d to 1500 mg/d because of increased transaminase levels, HPV-1 infection, or low neutrophil counts, within six months of initiating MMF treatment. These side effects were mild and symptomatic treatment were effective.

## Discussion

In this study, patients received MMF with concomitant low dose oral corticosteroids therapy in the first one to two years of MMF therapy. It was reported that the proportions of relapse-free patients that experienced improved ARR values and EDSS scores did not differ between patients treated with MMF alone and MMF in combination with prednisone [6]. Thus, we discuss the overall treatment of MMF with or without oral corticosteroids as MMF therapy. Similar to previous studies [3–10], MMF therapy significantly reduced ARR in 87% of patients, and 64% were relapse-free during a median course of 20 months (average 27) therapy with MMF. Our results confirmed that MMF therapy significantly decreased the risks of severe relapses, in terms of disability, as recently reported [9, 10, 13].

For patients receiving MMF therapy, EDSS scores at last follow-up were improved and maintained in 87% of the NMOSD patients, which is similar to the percentage of patients that experiencing improvement in their ARR. Hence, MMF is an effective treatment for reducing disabling relapses in NMOSD. We compared the disability status in remission before or at the beginning of MMF treatment with those at the last follow-up. This was done to minimize the influence on EDSS scores reductions caused by corticosteroid impulse therapy and spontaneous recovery in the acute phase. It was reported that 68% to 97% patients experienced improved or unchanged EDSS scores after MMF treatment [4, 9, 12, 14]. This difference in results may be caused by the varying number of the patients in acute phase and treatment duration. In our study, the post-treatment visual scores, which were not mentioned in any of the previous studies, remained statistically unchanged, suggesting that visual deficiency is usually fixed and more difficult to improve than the disability caused by myelitis.

As of now, there are no standard criteria for determining the optimal dosage of MMF for treating NMOSD among treatment centers. As this is a retrospective study based on a review of medical records, the evaluated NMOSD patients were receiving different daily doses of MMF. While all dosage levels of MMF have shown some benefit in reducing relapse rates in NMOSD patients, the lower and moderate dosage carry a much higher risk of relapse. Nearly 9 out of 10 of NMOSD patients taking the lower dosage of MMF experienced a relapse, which was reduced to 5 out of 10 patients in the moderate dosage group. These results verified that 1500 mg/d or less MMF was insufficient for most of the Chinese NMOSD patients. While there were no significant differences in EDSS between the three different treatment groups, longer follow-up times could have revealed different results. Hence, longer follow-up or prospective controlled trials are necessary to validate these findings. Considering that nearly 20% of the patients receiving 1750 to 2000 mg/d of MMF still relapsed, natural history studies infer a stepwise accumulation of attack-related disability for most patients with NMOSD and any treatment failure is potentially devastating for NMOSD patients [2]. Overall, we found that high-dose MMF therapy provided the most benefit to NMOSD patients.

One-third of our NMOSD patients had at least one coexisting systemic autoimmune disease. Any coexisting systemic autoimmune disease was a significant risk factor for relapse. As another point of view, MMF was also effective for Sjögren syndrome, systemic lupus erythematosus, and several other autoimmune disorders [15–17], suggesting that high-dosage MMF may be an optimal treatment for NMOSD patients with coexisting systemic autoimmune diseases. While most of the moderate to severe adverse effects were reported by the patients receiving high-dosage MMF (2000 mg/d) therapy, pharmacokinetic studies may allow for individualization of MMF dosing for NMOSD patients in the future.

In this study, the number of relapses before the initiation of MMF was another risk factor for further relapses in NMOSD patients. This indicated that patients were more prone to relapse if they had several prior attacks. The usage, dosage, and timing of prophylactic agents are still being actively investigated for NMOSD patients. However, these results suggest an adequate immunosuppressant should be recommended for NMOSD patients with a history of relapse.

In terms of tolerability, MMF was generally well tolerated in the 109 Chinese NMOSD patients. Adverse effects were reported by 21 patients, and 5 of the 52 patients receiving high-dosage MMF (2000 mg/d) reported moderate to severe adverse effects that required therapeutic intervention. From this study and other case series and cohort studies published so far [3–10], MMF was administrated alone or in combination with oral corticosteroids in more than 500 NMOSD patients. No serious toxicity concerns, such as malignancies or progressive multifocal leukoencephalopathy, which had been reported in transplant patients when MMF was used in conjunction with other immunosuppressants [14].

The present study was limited by its absent blinding, retrospective design, and the uneven assignment of patients to the different dosage group. Specifically, more recently diagnosed patients were more likely to receive the higher dosage of MMF. Despite these methodological limitations, this study provides useful information on the influence factors and optimal MMF dosage choice for the treatment of NMOSD. In the future, randomized controlled trials will be necessary to further verify the finds from this study.

## Conclusion

MMF is a safe and effective oral immunosuppressant for treatment of NMOSD. High-dosage MMF was more potent than the lower dose for treatment of NMOSD, with 1750 mg of daily MMF being the recommended dosage for Chinese patients with NMOSD. As for NMOSD patients with coexisting systemic autoimmune diseases, a higher daily dosage of MMF may be recommended.

## Additional file

Additional file 1: **Table S1.** Baseline demographic and clinical data of NMOSD patients receiving different dosages of MMF. There was no significant difference in baseline demographic and clinical data among the 3 groups of the NMOSD patients receiving different dosages of MMF. (DOC 41 kb)

## Abbreviations

ALT: Alanine aminotransferase
AQP4: Aquaporin-4
ARR: Annualized relapse rate
AST: Aspartate aminotransferase
EDSS: Expanded Disability Status Scale
HPV: Human papillomavirus
HR: Hazard ratio
IgG: Immunoglobulin G
MMF: Mycophenolate mofetil
NMO: Neuromyelitis optica
NMOSD: Neuromyelitis optica spectrum of disorders
ON: Optic neuritis
VA: Visual acuity
