# Supplementary material for: Dose effects of mycophenolate mofetil in Chinese patients with neuromyelitis optica spectrum disorders: a case series study
Source: BMC Neurol. 2018 Apr 23;18:47. doi: 10.1186/s12883-018-1056-x (PMC5911949; doi:10.1186/s12883-018-1056-x)
Supplement: Supplementary file 1 — Table S1. Baseline demographic and clinical data of NMOSD patients receiving different dosages of MMF. There was no significant difference in baseline demographic and clinical data among the 3 groups of the NMOSD patients receiving different dosages of MMF. (DOC 41 kb) [file 12883_2018_1056_MOESM1_ESM.doc]

**Table S1.** Baseline demographic and clinical data of NMOSD patients receiving different dosages of MMF

| **Characteristic** | **Patients with low dose MMF** | **Patients with moderate dose MMF** | **Patients with high dose MMF** | **P** |
| --- | --- | --- | --- | --- |
| **Number of Patients** | 11 | 23 | 52 |  |
| **Current age, median (range), y** | 49 (16-84) | 52 (15-77) | 53 (17-84) | 0.602 |
| **Female sex, No. (%)** | 8 (82%) | 20 (87%) | 48 (92) | 0.192 |
| **NMO diagnosis, No. (%)** | 7 (64%) | 17 (74%) | 40 (77%) | 0.683 |
| **Aquaporin 4 antibody positivity, No. (%)** | 9 (82%) | 20 (87%) | 47 (90%) | 0.703 |
| **Age at onset, median (range), y** | 43 (12-63) | 40 (10-63) | 43 (6-68) | 0.878 |
| **Disease duration before receiving MMF, median (range), mo** | 66 (12-258) | 68 (16-313) | 75 (6-535) | 0.968 |
| **Attack number before receiving MMF, median (range)** | 4 (2-15) | 4 (1-19) | 5 (1-33) | 0.547 |
| **Duration of MMF treatment, median (range), mo** | 25 (9-71) | 22 (6-89) | 18 (6-85) | 0.773 |
| **treatment-naïve patients, n (%)** | 2 (18%) | 9 (39%) | 13 (25%) | 0.355 |
| **ARR of Pre-MMF treatment, Median (Range)** | 1.2 (0.5-6) | 1.4 (0.2-6) | 1.5 (1.2-12) | 0.771 |
| **EDSS of Pre-MMF treatment, Median (Range)** | 4 (0-8.5) | 3 (0-6.5) | 3 (0-8.5) | 0.278 |
| **visual score of Pre-MMF treatment, Median (Range)** | 0 (0-9) | 2 (0-11) | 2 (0-10) | 0.695 |
